# Age-Related Variations in Serum Zinc Levels Among Female Patients in Sulaymaniyah, Iraq: Implications for Addressing Zinc Deficiency

**DOI:** 10.7759/cureus.42026

**Published:** 2023-07-17

**Authors:** Jihad M Hadi, Abdulqader A Al-Naqshbandi, Ardalan J Abdullah, Ayman M Mustafa, Karwan N Ghafar, Ayar M Hussain, Shapol S Rashid, Paiwast D Rasul

**Affiliations:** 1 Department of Nursing, College of Nursing, University of Human Development, Sulaymaniyah, IRQ; 2 Department of Pharmacy, College of Pharmacy, Hawler Medical University, Erbil, IRQ; 3 Department of Emergency Nursing, Haibat Sultal Technical Institute, Erbil, IRQ; 4 Medical Laboratory of Science, College of Health Sciences, University of Human Development, Sulaimaniyah, IRQ

**Keywords:** iraq, sulaymaniyah, age group, female, zinc deficiency

## Abstract

Background

Zinc plays a crucial role in human nutrition and various biochemical processes, making it indispensable for all life forms. Therefore, it is important to address low zinc levels, particularly among women, to prevent potential health issues.

Objective

This study aimed to evaluate the serum zinc levels of female patients in Sulaymaniyah, Iraq.

Methods

This retrospective cross-sectional study included a total of 299 patients, ranging in age from 16 to 48 years, who sought medical care at Baxshin Hospital in Sulaymaniyah Governorate, Iraq, between October 2022 and April 2023. The biochemical test was conducted to screen the patient's blood samples for serum zinc levels.

Results

Among 299 patients, 99 individuals had low zinc levels, 11 had high zinc levels, and 189 exhibited normal zinc levels. The analysis revealed a significant difference between low, normal, and high serum zinc levels, as evidenced by a p-value of <0.05. In terms of age-related variations, individuals under 20 years old had an average serum zinc level of 121.4 µg/dL. However, those between 21 and 30 years old demonstrated the highest average serum zinc level of 153.6 µg/dL, followed by 135 µg/dL for individuals aged 31-40, and 119 µg/dL for those above 40 years old.

Conclusion

These findings indicate that serum zinc levels may vary based on the age group of individuals. Consequently, further research is needed to explore the implications of these variations and establish appropriate strategies to address zinc deficiency among women in Sulaymaniyah.

## Introduction

Zinc is widely recognized as the most important trace element due to its various functions as a catalyst, structural element, and regulator in numerous metabolic processes. These processes include DNA transcription, gene expression, signal transduction, and endocrine function [1]. Zinc is essential for the healthy growth and development of various tissues in the body. More than 300 metal enzymes and 2,000 transcription factors rely on zinc as a cofactor for the synthesis of proteins, lipids, nucleic acids, and RNA. Additionally, zinc has effects that modulate the immune system and prevent apoptosis.

Zinc is thought to be the most important trace element because it plays a key role in many important metabolic processes such as DNA transcription, gene expression, signal transduction, and endocrine function [2]. It is necessary to consume zinc daily since there is no storage for it, and cellular access to zinc is regulated.

Zinc is relevant to metabolic disorders, such as insulin resistance, metabolic syndrome, and diabetes mellitus, for two main reasons. First, it plays a role in stabilizing the insulin hexamer [3,4]. Second, it acts as an antioxidant in oxidative stress, which is a primary factor in the development of insulin resistance and diabetes. The pancreas also stores zinc [5,6]. When intracellular zinc levels are lowered, insulin release is inhibited, leading to elevated blood sugar levels and increased vulnerability of Langerhans islet cells to damage. In diabetic individuals, another factor that may reduce zinc absorption is the consumption of white meat instead of red meat (to decrease the intake of saturated fatty acids) [7,8].

Gestational diabetes refers to glucose intolerance during pregnancy, which is caused by cellular dysfunction and insulin resistance. It typically starts in the second trimester and may lead to type 2 diabetes (T2DM) [9,10]. Inflammatory markers have been associated with the development of insulin resistance in gestational diabetes, although the results from different studies are conflicting. Variations in trace elements found in the serum of pregnant women have also been linked to gestational diabetes and glucose tolerance. Zinc deficiency can lead to complications such as low birth weight newborns, congenital abnormalities, and spontaneous abortions [11,12].

In women, zinc deficiency can result in various complications, including decreased synthesis and/or secretion of follicle-stimulating hormone (FSH) and luteinizing hormone (LH), abnormal ovarian development, disruption of the estrous cycle, frequent abortions, prolonged gestation, teratogenicity, stillbirths, difficulties in childbirth, pre-eclampsia, toxemia, and low birth weight of babies [13]. Most zinc transporters, metallothioneins, and metal regulatory transcription factors are expressed in oocytes, suggesting a key role for zinc in genome stability during early embryonic development [14].

Although overt zinc deficiency, as observed in certain medical conditions, is rare in human populations, marginal zinc deficiency may be quite common, even in industrialized nations [12,15]. Marginal zinc deficiency has been shown to contribute significantly to cell death caused by zinc depletion. Zinc supplementation restores antioxidant capacity and has been successful in treating conditions such as premature ejaculation and erectile dysfunction [16]. The aim of this study is to assess the levels of serum zinc in female patients of Sulaymaniyah, Iraq.

## Materials and methods

Materials and study design

This retrospective cross-sectional study included a total of 299 patients, ranging in age from 16 to 48 years, who sought medical care at Baxshin Hospital in Sulaymaniyah Governorate, Iraq, between October 2022 and April 2023. Male patients and other female nationalities, like females of Arab and Persian backgrounds, were excluded from the analysis. The study received ethical approval from Baxshin Research Center's ethics board (No. BRC 0201023) and was conducted in accordance with the principles outlined in the Declaration of Helsinki. Sample collection took place at Baxshin Hospital, utilizing a range of tools and equipment such as test tubes, Cobas-e411, racks, cotton swabs, disinfectants, syringes, compression bandages, and plasters.

Determination of serum zinc

Initially, a total of 299 serum samples were obtained from the patients for the purpose of measuring zinc levels. The measurements were performed using an automated device known as the Cobas e411, manufactured by Roche, Germany. The reference ranges used for interpreting the serum zinc levels were 70.6-114 μg/dL.

Statistical analysis

By calculating the frequencies and percentages, categorical variables were analyzed, and suitable statistical tests like Fisher's exact test or Pearson's χ2 test were used. Continuous variables were represented using either means and standard deviations or medians, depending on their distribution characteristics. The assessment of continuous data involved the utilization of either the Mann-Whitney U test or the student's t-test, depending on specific circumstances. The statistical analyses were conducted using IBM SPSS software version 26 (IBM Corp., Armonk, NY), with a significance level of 0.05 as the threshold for determining statistical significance.

## Results

The study focused on 299 female participants, categorized by age (under 20 to over 40 years old) and zinc status. The total number of females included in the study was 299, representing 100% of the participants. Among the 299 females, the distribution based on age groups was as follows: <20 years old (31 females, 10.4%), 21-30 years old (90 females, 30.1%), 31-40 years old (158 females, 52.8%), and >40 years old (20 females, 6.8%) (Table 1).

**Table 1:** Serum zinc level by age group for 299 patients

Age groups	Frequency (%) N= 299	Zinc level (mean μg/dL)
<20 years old	31(10.4)	121.4(76.9)
21-30 years old	90(30.1)	153.6(77.27)
31-40 years old	158(52.8)	135(80.29)
>40 years old	20(6.8)	119(80.12)

When the zinc levels were examined, the findings showed an increase in the subgroups of those under 20 (1 case, 0.33%), between 20 and 30 (2 cases, 0.67%), between 31 and 40 (7 cases, 2.34%), and over 40 (1 case, 0.33%). Age groups with normal zinc levels were those under 20 (18 instances, 6%), between 21 and 30 (58 cases, 19.4%), between 31 and 40 (100 cases, 33.4%), and above 40 (13 cases, 4.35%). Age groups with decreased zinc levels were those under 20 (12 cases, 4%), between 21 and 30 (30 cases, 10%), between 31 and 40 (51 cases, 17.1%), and above 40 (6 cases, 2%).

The results of the distribution of zinc status in terms of frequency and percentage were as follows: total (299 instances), high (11 cases, 3.7%), normal (189 cases, 63.2%), and low (99 cases, 33.1%) (Table 2).

**Table 2:** Demographic characteristics of serum zinc levels of patients that are in low, high, and normal levels

Age groups	Frequency (n)	Percent % (n / N * 100)
Decreased		
<20 years old	12	4
21-30 years old	30	10
31-40 years old	51	17.1
>40 years old	6	2
Total	99	33.1
Increased		
<20 years old	1	0.33
21-30 years old	2	0.67
31-40 years old	7	2.34
>40 years old	1	0.33
Total	11	3.7
Normal		
<20 years old	18	6
21-30 years old	58	19.4
31-40 years old	100	33.4
>40 years old	13	4.35
Total	189	63.2

**Figure 1:**
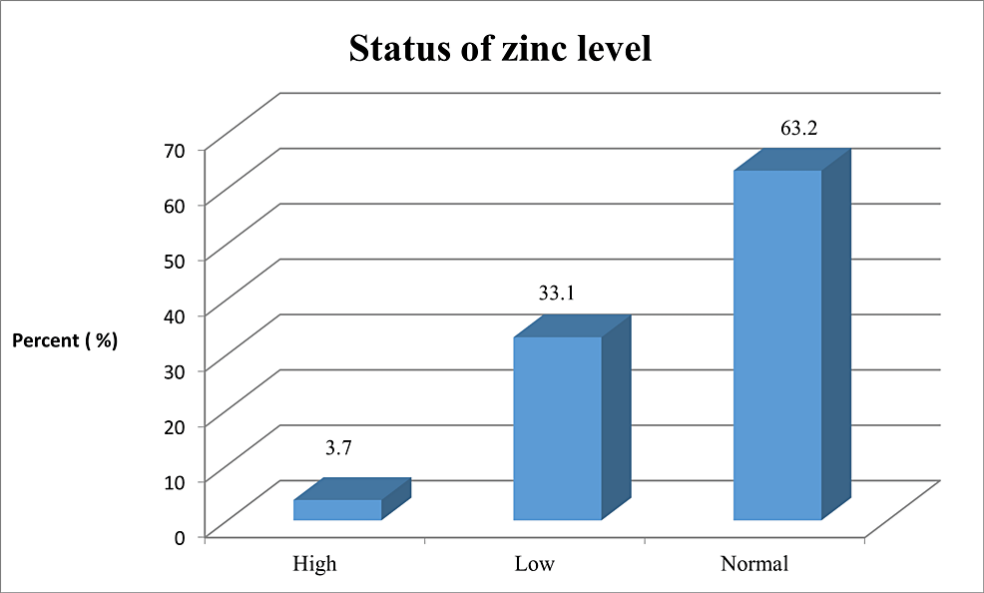
Status of zinc levels for all patients

Regarding the mean zinc levels, the results showed the following values for the different age groups: <20 years old (121.4 µg/dL, standard deviation 76.9), 21-30 years old (153.6 µg/dL, standard deviation 77.27), 31-40 years old (135 µg/dL, standard deviation 80.29), and >40 years old (119 µg/dL, standard deviation 80.12). The statistical analysis revealed a p-value of 0.000 (<0.05), t-stat of -0.33, multiple R of 0.22, and R square of 0.052 (Table 3). Figure 1 shows the status of zinc levels for all patients.

**Table 3:** P-value, T-test, multiple R, and R square of serum zinc for all patients

Age groups (years)	Patients (N=299)	zinc levels (μg/dL)	P-value	t-stat	Multiple R	R square
<20	31(10.4)	121.4(76.9)	0.000<0.05	-0.33	0.22	0.052
21-30	90(30.1)	153.6(77.27)				
31-40	158(52.8)	135(80.29)				
>40	20(6.8)	119(80.12)				

## Discussion

This retrospective cross-sectional study aimed to evaluate the serum zinc levels in Kurdish women residing in Sulaymaniyah, Iraq. The results revealed interesting findings regarding the distribution of zinc levels among different age groups and highlighted the prevalence of low zinc levels among the study participants.

Zinc is a crucial trace element involved in various metabolic processes and plays a vital role in DNA transcription, gene expression, signal transduction, and endocrine function [1]. The present study confirmed the importance of zinc by assessing its serum levels in a population of Kurdish women. It is worth noting that zinc deficiency can have significant health implications, particularly among women.

The global prevalence of zinc insufficiency is concerning, with approximately 17% of the population at risk. Certain groups, such as children, pregnant or nursing women, and the elderly, are particularly vulnerable to zinc deficiency [2]. In the context of pregnancy, low zinc levels can contribute to prolonged labor and increase the chances of preterm birth.

Extensive research has consistently demonstrated a strong link between zinc deficiency and conditions like overweight, obesity, and diabetes [3-5].

The findings indicated a significant difference between low, normal, and high serum zinc levels, with a p-value of less than 0.05. This suggests that factors other than age or gender may influence serum zinc levels in this population. However, further research is required to identify and explore these factors in detail.

In terms of age-related variations, the study observed interesting patterns in serum zinc levels among different age groups. Participants under 20 years old exhibited an average serum zinc level of 121.4 µg/dL, which increased to the highest level of 153.6 µg/dL in the 21-30 age group. Subsequently, the average serum zinc level decreased slightly to 135 µg/dL in the 31-40 age group and further declined to 119 µg/dL in individuals above 40 years old. These variations suggest that age may play a role in zinc metabolism and warrant further investigation.

The prevalence of low zinc levels among the study participants is a notable finding. Out of the 299 females included in the study, 99 individuals (33.1%) had low zinc levels. This highlights the vulnerability of Kurdish women residing in Sulaymaniyah to zinc deficiency. Zinc deficiency can have adverse effects on women's health, including disruptions in ovarian development, fertility issues, complications during pregnancy, and an increased risk of complications such as low birth weight, congenital abnormalities, and spontaneous abortions [11-13]. Interestingly, a study conducted in China, supported by the China Adult Chronic Disease and Nutrition Surveillance (CACDNS) in 2015, reported a lower prevalence of zinc deficiency among pregnant women (3.5%), suggesting a lower risk in that population [6]. A study from Spain found a strong correlation between increased gestational duration and lower serum zinc levels, indicating a potential impact of pregnancy on zinc status [7]. Similarly, pregnant Korean women commonly experience lower zinc levels (76.3%) due to the natural decline in zinc concentration during pregnancy [8].

However, it is essential to acknowledge the limitations of this study. The retrospective nature of the study and the relatively small sample size may limit the generalizability of the findings. Additionally, the study did not consider other potential confounding factors that could influence serum zinc levels such as dietary habits, socioeconomic status, and lifestyle factors. Future research should aim to address these limitations by conducting larger prospective studies that account for a more comprehensive range of variables.

Finally, the results of this study highlight the prevalence of low zinc levels and the potential impact of age on zinc metabolism. The findings underscore the importance of addressing zinc deficiency in this population to prevent potential health issues. Further research with larger sample sizes and more comprehensive methodologies is warranted to confirm these findings and develop effective strategies to address zinc deficiency among women in Sulaymaniyah.

## Conclusions

In conclusion, this study provides valuable insights into the serum zinc levels of female residents in Sulaymaniyah, Iraq, with a focus on different age groups. The results reveal significant variations in serum zinc levels across age groups, with individuals between the ages of 21 and 30 exhibiting the highest average levels. Furthermore, a considerable percentage of the female population in Sulaymaniyah has low serum zinc levels, highlighting the need for increased awareness and preventive measures. These findings serve as a foundation for future research to delve deeper into the relationship between age and serum zinc levels, as well as the potential health implications associated with these variables. It is crucial to evaluate serum zinc levels in women to ensure optimal nutrition and overall health.
